# Characteristics and progression of patients with frontotemporal dementia in a regional memory clinic network

**DOI:** 10.1186/s13195-020-00753-9

**Published:** 2021-01-08

**Authors:** Mélanie Leroy, Maxime Bertoux, Emilie Skrobala, Elisa Mode, Catherine Adnet-Bonte, Isabelle Le Ber, Stéphanie Bombois, Pascaline Cassagnaud, Yaohua Chen, Vincent Deramecourt, Florence Lebert, Marie Anne Mackowiak, Adeline Rollin Sillaire, Marielle Wathelet, Florence Pasquier, Thibaud Lebouvier, the Méotis network, Rachid Abied, Cathrine Adnet, Arnaud Barois, Stéphanie Baude, Véronique Berriot, Stéphanie Bombois, Gloria Boyer, Didier Brique, Gauthier Calais, Pascaline Cassagnaud, Hacène Drchekroud, Yaohua Chen, Joel Cliche, Charlotte Crinquette, Valérie Dachy, Valerie Debock, Anne Deprez, Vincent Deramecourt, Olivier Dereeper, Philippe Devos, Abdelghani Elazouzi, Adeline Enderle, Nicolas Fanjaud, Pierre Forzy, Karim Gallouj, Karine Garcon, Marie Honore, Dominique Huvent, Houria Idiri, Annabelle Ladeiro, Isabelle Lavenu, Florence Lebert, Thibaud Lebouvier, Patrick Le Coz, Eugénie Leclercq, Denis Lefebvre, Pierre Maciejasz, Marie-Anne Mackowiak, Rémi Messin, Florence Pasquier, Valérie Petit, Christine Plichon, Sandrine Ponthieu, Cécile Quievre, Jean Roche, Adeline Rollin Sillaire, Thierry Rosolacci, Olivier Senechal, Nathalie Taillez, Stéphanie Thibault Tanchou, Frédéric Tison, Sarah Tollot, Marie Trocmet, Charlotte Verpoort

**Affiliations:** 1 Univ. Lille, Inserm, CHU Lille, Lille Neuroscience & Cognition, CNRMAJ, LiCEND, DistAlz, F-59000 Lille, France; 2 Biostastitic department, CHU Lille, DistALz, Lille, France; 3 Univ. Lille, Inserm, CHU Lille, F-59000 Lille, France; 4 Sorbonne Université, Inserm U1127, CNRS UMR 7225, Institut du Cerveau (ICM), AP-HP - Hôpital Pitié-Salpêtrière, Paris, France; 5 Centre de référence des démences rares ou précoces, IM2A, Département de Neurologie, AP-HP - Hôpital Pitié-Salpêtrière, Paris, France; 6 Department of Public Health, CHU Lille, F-59000 Lille, France

**Keywords:** Frontotemporal dementia, Epidemiology, Progression, Dementia

## Abstract

**Background:**

Due to heterogeneous clinical presentation, difficult differential diagnosis with Alzheimer’s disease (AD) and psychiatric disorders, and evolving clinical criteria, the epidemiology and natural history of frontotemporal lobar degeneration (FTD) remain elusive. In order to better characterize FTD patients, we relied on the database of a regional memory clinic network with standardized diagnostic procedures and chose AD patients as a comparator.

**Methods:**

Patients that were first referred to our network between January 2010 and December 2016 and whose last clinical diagnosis was degenerative or vascular dementia were included. Comparisons were conducted between FTD and AD as well as between the different FTD syndromes, divided into language variants (lvFTD), behavioral variant (bvFTD), and FTD with primarily motor symptoms (mFTD). Cognitive progression was estimated with the yearly decline in Mini Mental State Examination (MMSE).

**Results:**

Among the patients that were referred to our network in the 6-year time span, 690 were ultimately diagnosed with FTD and 18,831 with AD. Patients with FTD syndromes represented 2.6% of all-cause dementias. The age-standardized incidence was 2.90 per 100,000 person-year and incidence peaked between 75 and 79 years. Compared to AD, patients with FTD syndromes had a longer referral delay and delay to diagnosis. Patients with FTD syndromes had a higher MMSE score than AD at first referral while their progression was similar. mFTD patients had the shortest survival while survival in bvFTD, lvFTD, and AD did not significantly differ. FTD patients, especially those with the behavioral variant, received more antidepressants, anxiolytics, and antipsychotics than AD patients.

**Conclusions:**

FTD syndromes differ with AD in characteristics at baseline, progression rate, and treatment. Despite a broad use of the new diagnostic criteria in an organized memory clinic network, FTD syndromes are longer to diagnose and account for a low proportion of dementia cases, suggesting persistent underdiagnosis. Congruent with recent publications, the late peak of incidence warns against considering FTD as being exclusively a young-onset dementia.

## Background

Frontotemporal lobar degeneration (FTD) is the second leading cause of early-onset dementia after Alzheimer’s disease (AD) [1]. FTD is characterized by changes in behavior and/or language due to the relatively selective atrophy of the frontal and temporal lobes [2]. In the past decade, the nosology of FTD has evolved outstandingly, prompting changes in diagnostic criteria. There are three main clinical presentations of FTD. The behavioral variant of FTD (bvFTD) is defined by an early and prominent behavioral and dysexecutive syndrome, whose core symptoms were revised by Rascovsky et al. in 2011 [3]. The two language variants of FTD (lvFTD) include the semantic and non-fluent presentations of primary progressive aphasia (PPA), also defined by updated clinical criteria [4]. In addition, FTD can initially present with motor symptoms (mFTD) such as features of atypical parkinsonism (progressive supranuclear palsy [PSP] and corticobasal syndrome [CBS]) [5].

Although being an umbrella term underlain by > 20 different possible pathologies [6], FTD stands as a unifying entity because of the lack of correlations between FTD syndromes and pathology [7]. bvFTD, for example, can be underlain by tau, TDP-43 or rarer pathologies, and on the contrary, one single pathology, such as PSP, can manifest with several clinical syndromes [6]. One exception to the unpredictability of the underlying pathology is the identification of a causal genetic mutation. Patients with FTD syndromes have a positive family history in 26–31% [8], highlighting the importance of genetics. The most common FTD mutations, all linked to a specific pathology, are found on *MAPT*, *PGRN*, and *C9ORF72* genes [8].

FTD prevalence was estimated between 0.01–4.61 per 1000 person and the incidence between 0.01–2.5 per 1000 person/year [9]. In recent dementia cohorts, FTD cases have been found to account for 1.6 to 7% of dementia cases [10, 11]. However, those figures need to be considered with caution. First, FTD is still underdiagnosed: neuropathological studies performed in communities where brain donation reaches a high level of acceptance show that as much as 5–9% of the elderly population with or without cognitive impairment at death has FTD pathology [12, 13]. It has been previously estimated that about 40% of FTD are misdiagnosed [14] and time to diagnosis is longer than for other dementias [15, 16]. Second, with some exceptions [17], most past estimations have been done using the previous Lund and Manchester [18] or Neary criteria [19]. Yet, the revised clinical criteria and the addition of new syndromes to the FTD spectrum outdate previous publications. Third, advances in neuropsychology, neuroimaging, and cerebrospinal fluid (CSF) biomarkers and genetics have improved FTD diagnosis in challenging situations such as psychiatric, amnestic, or late-onset presentations of the disease [20–22].

However, beyond research purposes, whether improving FTD diagnosis at the population level would stand a cost-benefit analysis is a subject that should be addressed open-mindedly. Indeed, one could argue that differential dementia diagnosis workup is a costly venture [23] that can be questioned in the absence of disease-modifying treatments. The demonstration that FTD diagnosis is associated with different prognoses and therapeutic approaches in routine care would advocate against a symptomatic approach of dementia.

Thus, data sharing on current FTD diagnoses and management is needed. We undertook the present study in a large regional memory clinic (MC) network to get a better overview of the incidence, characteristics and natural history of FTD syndromes defined using recent diagnostic criteria. The objectives were to study the characteristics of the FTD patients referred to the network from January 2010 to December 2016, including age at onset, time to diagnosis, clinical presentations, cognitive progression, and treatment.

## Patients and methods

### Patient selection

Founded in 1993, the Méotis network is the first French MC network, involving 30 MCs in the French Nord and Pas-de-Calais departments, sharing data within a common patient database since 1997. Meotis database reached a caseload of over 104,000 patients in 2018, representing more than 350,000 visits [24]. In all MCs, a multidisciplinary assessment is performed by neurologists, geriatricians, psychologists, dedicated nurses, and social workers; whenever necessary, patients can be assessed by psychiatrists, speech therapists, and dedicated nurses. Diagnostic work-up is harmonized throughout the network, and standardized data on patient characteristics and healthcare activity are systematically collected. All harmonized data are monitored and computerized by a data manager in the tertiary-referral Memory Resources and Research Center (MRRC) of the Lille University Hospital.

We included patients that were referred for the first time to one of the network’s MC from January 2010 to December 2016 and whose last clinical diagnosis during the follow-up was FTD, AD, or other causes of dementia. We first extracted all dementia cases to calculate the respective proportions of AD and FTD syndromes. Then, we focused on the subpopulation of AD and FTD syndromes for systematic comparisons. Since AD is the dominant cause of dementia, AD patients were chosen as a comparator. Data extraction was performed on September 2019, 33 months after the end of the inclusion period. For the few patients that received a diagnosis of bvFTD and lvFTD before the new criteria were published and were not followed up beyond 2011, we checked retrospectively that they fulfilled the revised diagnostic criteria. The bvFTD group comprised pure bvFTD [3] and a minority of patients with associated amyotrophic lateral sclerosis. The lvFTD group included a semantic and non-fluent agrammatic PPA [4] as well as rarer PPA variant such as apraxia of speech. The mFTD group comprised the PSP [25] and CBS [26] patients. Patients with overt motor neurone disease at presentation are usually not referred to our network because of a specialized regional amyotrophic lateral sclerosis care pathway.

### Data collection

We extracted the following data from the Méotis database: sex, age at first referral, referral delay, age at diagnosis, symptom onset, and diagnostic procedures. We collected the Mini Mental State Examination (MMSE) [27] and the short 4-item Instrumental Activities of Daily Living (IADL-4) [28] scores at first referral. In this article, IADLs score was calculated by summing up the number of maintained activities (ranging from 0 (full dependence) to 4 (complete autonomy)). The referral delay was defined as the interval, expressed in months, between symptoms onset (declared by the patient and caregiver) and first referral to the network. The clinical follow-up was defined as the interval, expressed in years, between the first and the last visit within the network. The survival was defined as the interval, expressed in years, between disease onset and death. Drug treatment was recorded at every visit. A patient was considered under a specific drug treatment if it was recorded at least once during follow-up.

Only the last clinical diagnosis was considered in this study because of its higher accuracy. The last diagnosis was the one made or kept after all diagnostic procedures and retained at follow-up. Diagnosis wandering was defined as the time from first referral to the last retained clinical diagnosis.

The date of death was retrieved from the National Institute of Statistics and Economic Studies (French: Institut national de la statistique et des études économiques) national death database thanks to the MatchID tool (https://deces.matchid.io/) on September 2020.

The datasets considered in the current study are available from the corresponding author on reasonable request. The database was declared to the ad hoc commission (Commission Nationale Informatique et Libertés (CNIL)) protecting personal data (#2146189 V1). Privacy and confidentiality rules were respected.

### Statistical analysis

Quantitative variables were described by the mean and standard deviation if the distribution was normal or by the median and interquartile range otherwise. Qualitative variables were described by the numbers and percentages of each modality.

Diagnostic subtypes (bvFTD, FTD mFTD and AD) were described and compared across all parameters. Quantitative variables were analyzed by an ANOVA or the Kruskal-Wallis non-parametric equivalent. Qualitative variables were compared by an exact chi-square or Fisher’s test (in the case of a theoretical number of cases below 5). A Bonferroni correction was applied to post-hoc comparisons of the FTD subgroups with respect to the AD group. The effect size was calculated as the standardized mean difference (for quantitative variables) and the Cramer’s V coefficient (for qualitative variables). A mixed linear model analyzed the evolution of the MMSE over time. The factors introduced into the model were time, diagnosis, and the interaction between diagnosis and time.

Incidence rates were calculated as the number of incident cases divided by the total number of person-years (py) for the catchment area over the 7 years. All rates were calculated using the reference population of the corresponding geographic area estimated by the French National Institute of Demographic Research (INED) on January 2015, as population at risk. Therefore, no variation was assumed during the 7 years of the study period. Age-standardized rates were calculated using the Revised European Standard Population 2013 (ESP2013). Results were presented in cases per 100,000 person-years.

Concerning mortality, median survival time after diagnosis was calculated for each diagnostic subtype, survival was estimated using the Kaplan–Meier model, and the log-rank test was used to test of differences in survival curves according to diagnostic subtype. Hazard ratios (HRs) were also adjusted for age and sex using Cox regression.

The analyses were performed using SAS software (version 9.4).

## Results

### Study population

Data from 26,525 demented patients followed in the network and fulfilling inclusion criteria were extracted. Among them, 2369 have first been seen at the MRRC (Lille tertiary-referral MC) and 24,156 first at one of the MCs belonging to the network.

During the 7 years of follow-up, 690 incident cases of FTD syndromes were identified, giving a crude incidence rate of 2.42 per 100,000 person-years (Table 1). The FTD incidence across age groups at diagnosis reached its peak in the 75-to-79 year-old group, with an incidence rate of 14.95 per 100,000 person-years. The age-standardized incidence rate was 2.90 per 100,000 person-years.

**Table 1:** FTD incidence rates, number of cases, and number of person-years by age group

Age group at diagnosis	Number of cases	Number of person-years	Incidence (per 100,000 person-years)
0–39	2	14,854,742	0.01
40–44	3	1,922,550	0.16
45–49	8	1,842,470	0.43
50–54	25	1,862,287	1.34
55–59	61	1,796,207	3.40
60–64	95	1,703,044	5.58
65–69	123	1,490,146	8.25
70–74	110	828,709	13.27
75–79	126	842,548	14.95
80–84	92	725,613	12.68
≥ 85	45	676,866	6.65
Total	690	28,545,202	2.42

**Fig. 1:**
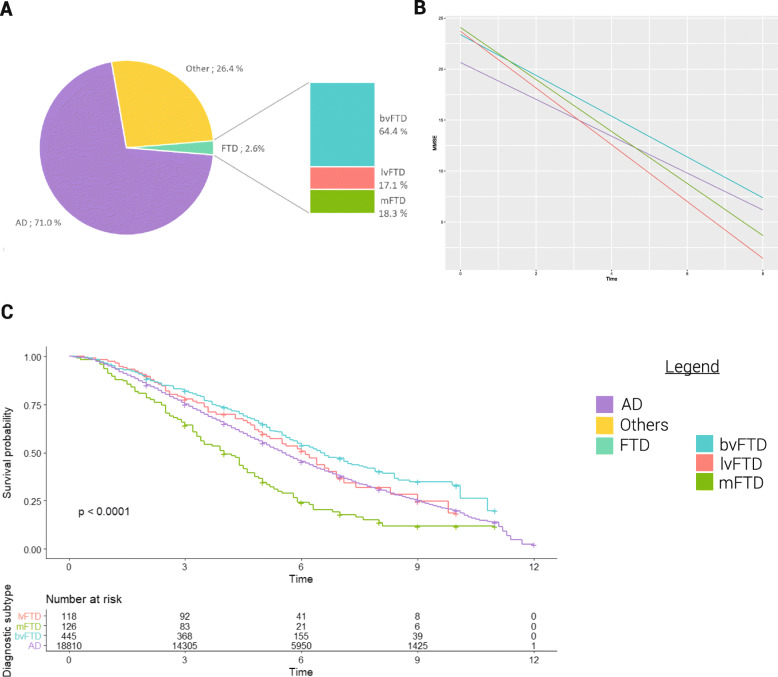
Characteristics and progression of patients with FTD syndromes in the Méotis network (incident cases from 2010 to 2016). **a** Etiologies of dementia in the incident cases. **b** Mixed linear model of the evolution of the MMSE over time in patients with FTD syndromes and AD. **c** Survival in patients with FTD syndromes and AD. *AD*, Alzheimer’s disease; *FTD*, frontotemporal dementia; *bvFTD*, behavioral variant of the frontotemporal dementia; *lvFTD*, speech variant of the frontotemporal dementia; *mFTD*, motor variant of the frontotemporal dementia; Other, other type of dementia due to a neurodegenerative or vascular disease

FTD syndromes represented 2.6% of the studied population, as compared with 71% AD (Fig. 1a). Among FTD syndromes, 64% were bvFTD, 17% lvFTD, and 18% mFTD (Table 2). The proportion of FTD syndromes was higher in the MRRC (8.1%) than elsewhere (2.0%).

**Table 2:** Demographics and clinical features of FTD syndromes

	bvFTD			lvFTD			mFTD			AD	
	***N*** = 446			***N*** = 118			***N*** = 126			***N*** = 18,831	
Variable	Value	NA	p*	Value	NA	p*	Value	NA	p*	Value	NA
Women, *n* (%)	232 (52.2)	0	**< 0.001**	68 (57.6)	57.6	**0.01**	68 (54)	0	**< 0.001**	13,162 (69.9)	0
Age at first visit, m (sd)	69.4 (10.3)	0	**< 0.001**	72.7 (9.5)	0	**< 0.001**	72.0 (8.0)	0	**< 0.001**	80.6 (7.5)	0
Age at diagnosis, m (sd)	70.2 (10.3)	0	**< 0.001**	73.6 (9.7)	0	**< 0.001**	72.8 (8.1)	0	**< 0.001**	81.0 (7.3)	0
Delay referral (months), m (sd)	40.0 (41.2)	87	**< 0.001**	30.8 (20.5)	19	0.84	35.8 (29.9)	24	0.13	31.8 (32.0)	5370
Diagnosis wandering (months), m (sd)	9.9 (16.8)	0	**< 0.001**	10.5 (16.4)	0	**< 0.001**	9.1 (15.1)	0	**< 0.001**	5.8 (14.2)	0
Clinical follow-up (months), m (sd)	25.2 (24.0)	0	**< 0.001**	24.2 (22.0)	0	**< 0.001**	20.2 (19.6)	0	0,05	17.5 (21.4)	0
Dementia family history, *n* (%)	75 (16.8)	0	**< 0.001**	11 (9.3)	0	**< 0.001**	13 (10.3)	0	**< 0.001**	508 (2.7)	0
MMSE at first visit, m (sd)	22.1 (6.1)	117	**0.02**	20.6 (7.5)	27	0.002	21.9 (6.1)	38	**< 0.001**	18.9 (6.0)	2246
IADL-4, median	3	199	**< 0.001**	4	38	**< 0.001**	3	54	**< 0.001**	2	6689
PET in the network, *n* (%)	117 (26.2)	0	**< 0.001**	18 (15.2)	0	**< 0,001**	25 (19.8)	0	**< 0.001**	484 (2.5)	0
CSF in the network, *n* (%)	135 (30.3)	0	**< 0.001**	22 (18.6)	0	**< 0,001**	33 (26.2)	0	**< 0.001**	759 (3.9)	0
Death, *n* (%)	213 (48.4)	0		63 (53.4)	0		96 (76.2)	0		11,220 (59.1)	0
Survival in years, median	6.5	0	0.18	6.1	0	0.14	4.0	0	**< 0.001**	5.5	0
Autopsy verification, *n* (%)	4 (0.8)	0		0 (0)	0		5 (4.0)	0		6 (0.03)	0

*AD* Alzheimer’s disease, *FTD* frontotemporal dementia, *bvFTD* behavioral variant of the frontotemporal dementia, *IADL* Instrumental Activities of Daily Livings, *lvFTD* speech variant of the frontotemporal dementia, *mFTD* motor variant of the frontotemporal dementia, *IADL-4* Instrumental Activities of Daily Living—4, *MMSE* Mini Mental State Examination, *m (sd)* mean (standard deviation), *PET* position emission tomography, *CSF* cerebrospinal Fluid. *Comparison to AD

### Characteristics of patients with FTD syndromes

The sex ratio significantly differed between AD and FTD patients (*p* <  0.0001, *d* = 0.1) (Table 2). Men represented 47% of FTD and only 30% of AD patients. Patients with FTD syndromes were younger than those with AD at first referral (70.4 vs. 80.6 years, *p* <  0.0001), and bvFTD patients were younger than the remaining FTD syndromes (69.4 vs. 72.3 years).

MMSE scores at first referral were higher in FTD syndromes than in AD (21.8 vs. 18.9, *p* <  0.0001, *d* = 0.5). Likewise, the median IADL-4 score was higher in patients with FTD syndromes compared to AD patients (3 vs. 2, *p* <  0.0001, *d* = 0.5), favoring a more preserved autonomy in instrumental activities.

Among the FTD syndromes, a positive family history of dementia was identified in 14%, as compared with 2.3% of AD patients (*p* < 0.0001, *d* = 0.1). Among the 294 FTD syndromes referred to the MRRC, a genetic mutation was detected in 34% of the 99 patients in whom the genetic analysis was performed (47% in *C9Orf72*, 32% in *PGRN*, and 21% in *MAPT* genes). Mutations were more likely to be retrieved in bvFTD (95%) than in lvFTD (5%) or mFTD (0%). See Table 1 for detailed comparisons between FTD syndromes and AD.

### Diagnosis of FTD syndromes

We then systematically studied the time to referral, time to diagnosis and diagnostic workup of FTD compared to AD patients. Referral delay was longer for FTD syndromes compared to AD (37.6 vs. 31.8 months, *p* < 0.0001, *d* = 0.4). Among the FTD syndromes, referral delay was the highest for bvFTD (40.0 vs. 33.3 months in other FTD). Diagnosis wandering was longer for FTD syndromes compared to AD (9.8 vs. 5.8 months, *p* < 0.001, *d* = 0.1), but similar across FTD syndromes.

As part of the standardized dementia diagnosis procedure, all of our patients performed an MRI, if not contraindicated. The diagnostic workup of FTD patients in the whole Méotis network included more often a FDG-PET and a lumbar puncture that the one of AD patients (23.2% vs. 2.6% and 27.5% vs. 3.96% respectively, *p* < 0.001 and *d* = 0.2 for both comparisons, Table 2). Brain imaging and lumbar puncture were more consistently used in Lille MMRC both for AD and FTD diagnosis (our unshown data).

Correlations between clinical diagnoses and pathology were excellent in the 15 patients of the study population who came to autopsy. Among the patients with available pathological examination, the 4 in the bvFTD group had FTLD-TDP (*n* = 3) or FTLD-FUS (*n* = 1) pathologies. All 5 patients in the mFTD group had PSP or CBD pathology. All 6 patients in the AD group had AD pathology +/− cerebral amyloid angiopathy or Lewy body pathology (*n* = 3 and 2, respectively).

### Natural history of FTD syndromes

Cognitive progression estimated by the rate of MMSE decline was then assessed. Overall, there was no significant difference in the rate of MMSE decline between FTD syndromes and AD. Across FTD syndromes, bvFTD did not significantly differ from AD in the rate of MMSE decline per year (the slope was *a*_bvFTD_ = − 2.0 in bvFTD against *a*_AD_ = − 1.8 in AD, *p* = 0.4). However, the decline was higher in lvFTD (*a*_lvFTD_ = − 2.8) and mFTD (*a*_mFTD_ = − 2.6) than in AD patients (*p* = 0.003 and *p* = 0.02, respectively) (Fig. 1b). Follow-up was longer for FTD syndromes compared to AD (24.1 vs. 17.5 months, *p* < 0.0001, *d* = 0.2), and more specifically, bvFTD and lvFTD patients had a significantly longer follow-up than AD patients.

As of September 2020, 48% of bvFTD, 53% of lvFTD, 76% of mFTD, and 59.1% of AD patients had died (Table 2). The median survival time after diagnostic was 5.5 years for the entire sample and varied significantly according to the diagnosis subtype (6.5 years for bvFTD, 6.1 for lvFTD, 5.5 for AD, and 4.0 for mFTD, *p* < 0.001) (Fig. 1c). Age (HR [CI 95%] = 1.05 [1.05–1.06] for 1 year, *p* < 0.001) and male sex (1.73 [1.67–1.80], *p* < 0.001) were significantly associated with an increased risk of death. After adjustment for age and sex, mFTD were significantly associated with a lower median survival as compared to AD (2.32 [1.89–2.84], *p* < 0.001). There were no significant differences between AD and bvFTD (1.10 [0.96–1.26], *p* = 0.179) and between AD and lvFTD (1.21 [0.94–1.55], *p* = 0.137).

### Treatment of FTD syndromes

**Fig. 2:**
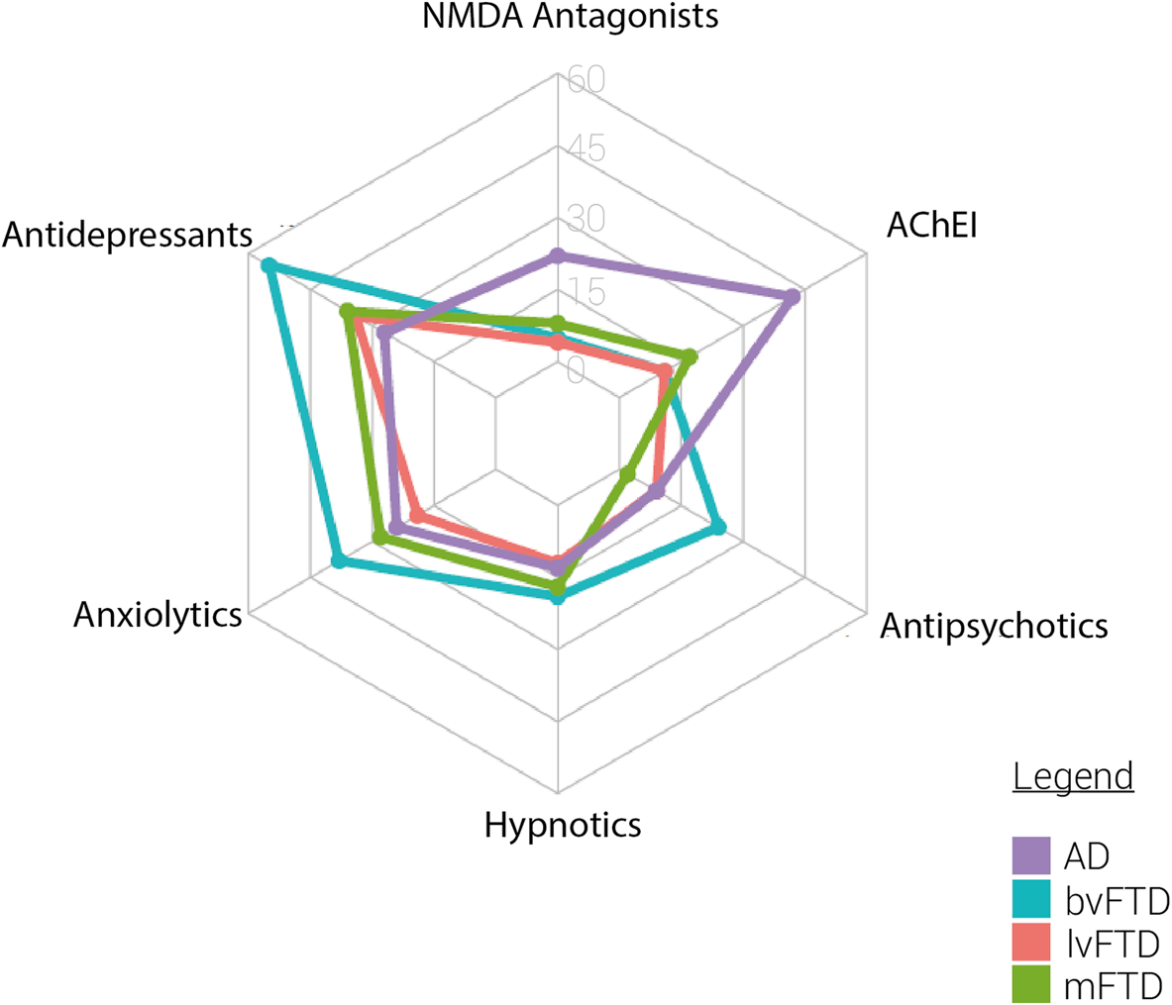
Drug treatments used in FTD syndromes compared to AD. AD, Alzheimer’s disease; AChEI, anticholinesterase inhibitor; bvFTD, behavioral variant of the frontotemporal dementia; lvFTD, speech variant of the frontotemporal dementia; mFTD, motor variant of the frontotemporal dementia

There were sharp differences in the therapeutic approach between FTD syndromes and AD. FTD patients received less anticholinesterase inhibitors (AChEI) and memantine than AD patients (12.0% vs. 42.2%, *p* < 0.0001, *d* = 0.1 and 5.7% vs. 21.8%, *p* < 0.0001, *d* = 0.1, respectively). Conversely, FTD patients received more antidepressants (48.0% vs. 27.0%, *p* < 0.0001), anxiolytics (33.2% vs. 23.6%, *p* < 0.0001, *d* = 0.04), and antipsychotics (17.5% vs. 13.1%, *p* = 0.003, *d* = 0.1) than AD patients. The difference between AD and FTD stemmed mostly from the bvFTD group where antidepressants (55.2%), anxiolytics (38.3%), and antipsychotics (24.4%) were used the most (*p* < 0.0001 and *d* = 0.1 for the three comparison to AD). There was no significant difference in the use of hypnotics between groups (Fig. 2).

## Discussion

The main findings of the present study are threefold: (1) despite new sets of criteria, diagnoses of FTD syndromes remained low in routine care in our regional memory clinic network; when diagnosed, bvFTD patients had longer referral delay and diagnostic wandering than AD patients; (2) the peak of incidence of bvFTD occurred between 75 and 79 years, clearly advocating against the conception of FTD as exclusively an early-onset dementia; (3) FTD syndromes differed from AD with regard to cognition and autonomy at baseline, cognitive decline, and disease duration; and (4) therapeutic strategies radically differed from the ones in AD.

### Misconceptions about FTD lead to underdiagnosis

In this retrospective study, we calculated an FTD age-standardized incidence rate of 2.9/100.000 py in our region. Our results stand in-between the ones of two recent studies using updated FTD criteria that found an incidence of 1.6/100.000 py in the UK (Norfolk and Cambridgeshire counties) and 3.05/100.000 py in Italy (Leccia and Brescia provinces) [17, 29]. However, while we used the same European reference population as our British colleagues, Logroscino et al. used the Italian population for standardization. Standardization of their incidence rate with the same European population yields an FTD age-standardized incidence rate of 2.78/100.000 py, strikingly similar to ours (*our unshown data*).

We found that FTD syndromes represented 3% of the Méotis network caseload. Similar MC surveys in Netherlands [11] and Sweden [10] had 7% and 3.6% of FTD syndromes, respectively. However, all patients in the Dutch cohort were followed in the Alzheimer center of the VU University Medical Center (VUmc), a tertiary center where atypical dementias are likely to be addressed, possibly leading to an overrepresentation of FTD patients. Likewise, there was a 8.1% proportion of FTD patients in Lille tertiary center. A recent review on the epidemiology of FTD highlighted three studies with high methodological standards [9]. In these publications using the Lund and Manchester [18] or Neary [19] criteria, FTD syndromes accounted for 1.1% [30], 3% [31], and 3.8% [32] of dementia cases, which is consistent with our findings.

In sharp contrast, consistent with the underdiagnosis of FTD, systematic neuropathology surveys show much higher figures. In UK brain banks from donors (of whom two thirds had dementia), FTD represented 5.1% of diagnoses [33] and up to 9.4% of elderly people participating in a community brain donation program were found to have some FTD lesions at autopsy [13].

The reasons for FTD underdiagnosis are manifold. First, late-onset FTD are often overlooked. FTD is historically considered as a major cause of early onset dementia [1], which probably contributes to FTD diagnosis being overlooked in late-onset dementia. Yet, in recent studies with pathological confirmation, one fourth of FTD cases had an age at onset > 65 years [34]. In the recent literature, there is a trend toward an increase in the age at diagnosis of FTD syndromes, which may relate to the increasing age at dementia diagnosis in recent surveys [24]. While older studies showed an age at diagnosis of 65.9 years [35], we found an age at diagnosis of 71.3 years, which compares to recent publications showing a mean age at diagnosis of 69.4 [36], 70.0 [37], or 71.3 years [29]. Interestingly, the peak of incidence occurred between 75 and 79 years in our survey as in the aforementioned Italian and English studies [17, 29], reminding that FTD is not only a dementia of early onset.

Second, the positive diagnosis of bvFTD and its differentiation with primary psychiatric disorders is another diagnostic challenge [38] that is reflected by the increased time to presentation and time to diagnosis of the bvFTD variants as compared with the others [39, 40]. Prolonged diagnostic wandering in bvFTD, associated in our study with an increased reliance on diagnostic biomarkers, seems to be a universal finding [15–17] and suggests that many cases could remain misdiagnosed. Future studies should focus on the exact determinants of the delay in referral and in diagnosis. Third, all the possible clinical presentations of FTD have not been thoroughly described and some are not taken into account by the available clinical criteria. The amnestic variant of FTD, in particular, is difficult to differentiate from AD [20, 41] in particular in late-onset dementia [42]. Another example is the right temporal variant of FTD—although a recent publication proposing clinical criteria will contribute to fill the gap [43].

Overall, our survey confirms that FTD are still probably overlooked despite the use of novel clinical criteria and incorporation of new phenotypes. While progress has been made in the recognition of late-onset forms, differential diagnosis between FTD and AD remains a challenge, particularly in the oldest old, and bvFTD cases are probably still mistaken for primary psychiatric disorders.

### FTD syndromes differ with AD in baseline characteristics and natural history

We found several key differences between FTD syndromes and AD at baseline. First, as we had previously shown [39], we confirmed that the MMSE score is higher in FTD. However, behavior, social cognition, and executive functions, the main domain impaired in bvFTD, are not properly assessed by the MMSE, which somewhat undermines the assumption that the general cognitive status is better preserved in FTD syndromes. The higher IADL-4 score in FTD compared to AD contrasted with past studies that retrieved either lower [44] or equal [45] autonomy. However, IADL-4 only assesses restriction in four activities (telephone, transportation, drug treatment, and finances) that are best associated with future dementia risk [28], thus preventing a direct comparison of our results with studies that employed the full ADL. The younger age and the better preservation of memory and visuo-motor functions may explain the lesser impairment found in FTD as compared to AD. Impaired functional capacity in bvFTD is primarily due to behavioral symptoms and impaired social cognition, and the routine (although complex) instrumental activities of the IADL-4 may not be the most representative of the loss of autonomy in FTD syndromes. Among the FTD syndromes, the lvFTD patients had the most preserved autonomy, as found in previous studies [44, 45].

Although FTD syndromes as a whole had a similar rate of MMSE decline to AD, lvFTD and mFTD variants specifically showed a higher rate of MMSE decline in time. Additionally, lvFTD had a slightly lower score at baseline than the other variants. Since the MMSE relies mostly on language, aphasia has likely impacted the score in lvFTD. In recent studies, patterns of longitudinal MMSE decline across the FTD phenotypes have already been studied, and semantic dementia cases were shown to decline the most [46]. Regarding survival, we, as others [17], reported that mFTD had the more severe prognosis of FTD syndromes [17], followed by lvFTD and bvFTD. Despite similar MMSE decline rates between bvFTD and AD, mFTD patients had a significantly lower survival median.

### Therapeutic strategies in FTD

The drug treatments used in FTD syndromes markedly differed from the ones used in AD. These observations should be interpreted with caution since differences may only reflect different customs, and not different responses to treatment. However, clinical guidance on the symptomatic treatment of FTD is limited [47], prompting physicians to use psychotropic drugs that may be used non-specifically in dementia, based on the medical needs and immediate efficacy. Hence, the prescription habits in FTD may also reflect the neuropsychiatric symptoms and treatment response of FTD patients.

A publication from the Boxer’s group showed that off-label use of AChEI and memantine in FTD was common in the USA in 2010 [48]. In our region and in the 2010–2019 time span, we found that AChEI and memantine were used in only 12.0% and 5.7% of FTD syndromes, in accordance with recent data supporting lack of efficacy—or even deleterious effects in bvFTD and mFTD ([49, 50], reviewed in [51, 52]). The remaining prescriptions may reflect diagnostic hesitations with AD at the beginning of follow-up.

Antipsychotics and anxiolytics were more frequently used in FTD syndromes than in AD, and the difference with AD was driven by the bvFTD variant. Antipsychotics are prescribed to treat agitation in dementia whatever the etiology (AD, FTD, or others) [53], although their use is restricted to patients with severe symptoms (aggression, agitation, or psychosis) who fail to respond adequately to other pharmacological and nonpharmacological treatments. The use of anxiolytics and antipsychotics in 38.3% and 24.4% of bvFTD patients, as opposed to 23.6% and 9.3% in AD, is thus a reflection of the higher rate of productive behavioral symptoms (e.g., agitation, aggression, and psychosis) in this variant. However, the low rate of antipsychotics use in FTD demonstrated that physicians took into account the alerts on side effects [54, 55] and increased mortality rate [56] in FTD and dementia patients treated with antipsychotics. The black box warning from the Food and Drug Administration was followed by a similar warning from the French Haute Autorité de Santé in 2009 (https://www.has-sante.fr/jcms/c_885227/fr/limiter-la-prescription-de-neuroleptiques-dans-la-maladie-d-alzheimer) that had found a strong echo in the neurologic and geriatric communities.

The most remarkable difference however regarded the prescription of antidepressants, which was twice as important in bvFTD (55.2%) as in AD (27.0%). Indeed, although results are mixed, comprehensive reviews of the evidence from clinical trials favored the use of selective serotonin reuptake inhibitors to treat behavioral symptoms [47, 51, 52, 57]. Our team in particular demonstrated that trazodone, a serotonin antagonist and reuptake inhibitor, reduced irritability, agitation, and depressive symptoms in FTD [58]. The much better tolerance profile and apparent efficacy of serotonin-acting drugs logically imposed them as the mainstay of FTD treatment in our network.

### Strengths and limitations

This naturalistic study of a 6-year period is rooted in 23 years of data sharing and harmonization across a regionwide memory clinic network [24]. It allowed to analyze the trends of real-life FTD diagnosis and care over time. We reached a considerable number of new patients per year, equivalent to the one of nation-wide MC networks. Analyzing the characteristics of consecutive FTD patients first referred between 2010 and 2016 allowed us to focus on patients in which the diagnosis was made using the new criteria for bvFTD and lvFTD [3] and strengthened by follow-up. By considering a wide spectrum of FTD variants, we included patients that are often withdrawn from FTD cohorts.

Our survey confirmed many previously published data, which reinforces of the quality and validity of our database. We showed that approximately two thirds of FTD patients had a behavioral variant (bvFTD), and 17% had a language variant, which matches other databases [9, 59, 60]. We, as others, found a sex ratio of approximately 1:1 in FTD [9]. Thirty-five percent of our FTD patients had a family history of neuropsychiatric disease, in agreement with the literature [14, 61]. Only our rate of mutations was lower than previously reported since a mutation was identified in *C9ORF72*, *MATP*, or *GRN* in only 6% of the FTD patients that had a genetic analysis, against 10–15% in the literature [62]. Last but not the least, pathological diagnoses when available matched the clinical diagnoses, confirming the high accuracy of the clinical diagnoses made in a structured regional network and confirmed by a prolonged follow-up.

Our survey has however a few limitations. First, important data are not systematically populated in our database. We still lack accurate cognitive, functional, or disease-specific scales to assess disease progression. Furthermore, the mean follow-up of ~ 2 years precludes a comprehensive overview of FTD progression in many of our patients. We also acknowledge a selection bias due to the different networks involved in movement disorders and dementia care in our region, an issue that had been acknowledged in similar studies. Patients with overt motor neuron disease at presentation were not included because they were referred to a specialized regional care pathway rather than to memory clinics. Likewise, the PSP and CBS patients that were referred to our memory clinics were probably the ones presenting early behavioral and/or cognitive changes. Conversely, PSP and CBS with prominent motor symptoms were likely to be followed in movement disorders clinics, where secondary referral to MCs is not systematic. Still, our incidence rate compares to the ones of two regional cohorts including the full FTD spectrum [17, 29].

### Conclusion and outlook

Overall, our study showed that FTD syndromes have specific clinical features, different progression patterns, and therapeutic strategies. Yet, even in a region with an organized memory clinic network, FTD is still overlooked and diagnosis wandering remains longer than in AD. Psychiatric, amnestic, and/or late-onset presentations of FTD are particularly treacherous, and the overlap between cognitive/behavioral and motor presentations leads to an underestimation of the motoric presentations of FTD in memory clinics.

There is an obvious need of accurate FTD biomarkers to improve FTD diagnosis. Until and even after the avenue of such biomarkers, neuropsychology has and will have a role to play at a limited cost. The development of novel tests exploring new domains of social cognition beyond mentalization and emotion recognition is a steppingstone in this direction. Social cognition deficits have been found to be a reliable and effective cognitive marker of FTD, especially in patients with a psychiatric [63] or amnestic [64, 65] presentations. Social cognition deficits are probably underestimated in mFTD as well [66], advocating for a more systematic assessment of social cognition in memory, geriatric, movement disorders, and psychiatry clinics. In order to improve FTD diagnosis, the classical boundaries between specialties should be broken. Indeed, it is only through a harmonization in diagnostic procedures and databases involving geriatricians, movement disorders specialists, old-age psychiatrists, neuropsychologists, speech-therapists, and memory clinics that the real scope of FTD will be thoroughly apprehended. The gathering of these different disciplines into consortiums such as the Centers of Excellence in Neurodegeneration (CoEN) responds to this objective.

Additionally, initiatives are needed to raise awareness on FTD in the general population. At the eve of disease-modifying therapies, misdiagnosis of FTD may already be a loss of opportunity for patients.

## Data Availability

The datasets considered in the current study are available from the corresponding author on reasonable request.

## Abbreviations

AD: Alzheimer’s disease
AChEIs: Acetylcholinesterase inhibitors
ANOVA: Analysis of variance
bvFTD: Behavioral variant of frontotemporal dementia
C9ORF72: Chromosome 9 open reading frame 72
CBD: Corticobasal degeneration
CBS: Corticobasal syndrome
CSF: Cerebrospinal fluid
FTD: Frontotemporal dementia
IADL: Instrumental Activities of Daily Living
MAPT: Microtubule-associated protein tau
MC: Memory Clinic
mFTD: Frontotemporal lobar degeneration with primarily motor symptoms
MMSE: Mini mental state examination
MRRC: Memory Resources and Research Center
lvFTD: Language variants of frontotemporal lobar degeneration
PPA: Primary progressive aphasia
PGRN: Progranuline
PSP: Progressive supranuclear palsy
TDP-43: TAR DNA-binding protein 43
